# Comprehensive gene expression analysis for exploring the association between glucose metabolism and differentiation of thyroid cancer

**DOI:** 10.1186/s12885-019-6482-7

**Published:** 2019-12-30

**Authors:** Hoon Young Suh, Hongyoon Choi, Jin Chul Paeng, Gi Jeong Cheon, June-Key Chung, Keon Wook Kang

**Affiliations:** 1 Department of Nuclear Medicine, Seoul National University Hospital, 28 Yongon-Dong, Jongno-Gu, Seoul, 03080 Republic of Korea; 2 Department of Nuclear Medicine, National Cancer Center, 323 Ilsan-ro, Ilsandong-gu, Goyang-si, Gyeonggi-do 10408 Republic of Korea

**Keywords:** Glucose metabolism, Thyroid cancer, Tumor differentiation, Glycolysis, GLUT

## Abstract

**Background:**

The principle of loss of iodine uptake and increased glucose metabolism according to dedifferentiation of thyroid cancer is clinically assessed by imaging. Though these biological properties are widely applied to appropriate iodine therapy, the understanding of the genomic background of this principle is still lacking. We investigated the association between glucose metabolism and differentiation in advanced thyroid cancer as well as papillary thyroid cancer (PTC).

**Methods:**

We used RNA sequencing of 505 patients with PTC obtained from the Cancer Genome Archives and microarray data of poorly-differentiated and anaplastic thyroid cancer (PDTC/ATC). The signatures of GLUT and glycolysis were estimated to assess glucose metabolic profiles. The glucose metabolic profiles were associated with tumor differentiation score (TDS) and BRAFV600E mutation status. In addition, survival analysis of glucose metabolic profiles was performed for predicting recurrence-free survival.

**Results:**

In PTC, the glycolysis signature was positively correlated with TDS, while the GLUT signature was inversely correlated with TDS. These correlations were significantly stronger in the BRAFV600E negative group than the positive group. Meanwhile, both GLUT and glycolysis signatures were negatively correlated with TDS in advanced thyroid cancer. The high glycolysis signature was significantly associated with poor prognosis in PTC in spite of high TDS. The glucose metabolic profiles are intricately associated with tumor differentiation in PTC and PDTC/ATC.

**Conclusions:**

As glycolysis was an independent prognostic marker, we suggest that the glucose metabolism features of thyroid cancer could be another biological progression marker different from differentiation and provide clinical implications for risk stratification.

**Trial registration:**

Not applicable.

## Background

Molecular imaging has been widely used for the biological characterization of thyroid cancer in the clinic. Iodine and glucose metabolism have been respectively identified by radioactive iodine imaging and 2-deoxy-2-[^18^F]fluoro-D-glucose (FDG) positron emission tomography (PET), which have played important role in therapeutic plan [1, 2]. The fact that thyroid cancers with poor differentiation have lower I-131 avidity and higher F-18 FDG avidity have long been used to presume the aggressiveness of thyroid cancer [3]. This ‘flip-flop phenomenon’ was thought to have resulted from the loss of I-131 concentration capacity with increasing demand for glucose during the dedifferentiation of tumor cells [4].

As metabolic features of thyroid cancer could be associated with genomic alteration as well as tumor differentiation, previous studies have revealed the trend of relationship by using imaging studies and pathologic profiles [3, 5, 6]. Among the major cancer drivers of differentiated thyroid cancers, B-type Raf kinase (BRAF) mutation exclusively occurred in papillary thyroid cancer (PTC) and PTC-derived anaplastic thyroid cancer (ATC) is associated with low iodine avidity [3]. Thyroid cancer with BRAF mutation acquires more aggressive features through BRAF mutation-associated silencing of thyroid-specific genes, differentiation markers of iodine metabolism [6]. The dedifferentiation according to BRAF mutation leads to increased glucose consumption of cancer cells [1], which is supported by the finding that PTC with BRAF mutation showed a trend of more FDG avidity [7]. In spite of these cross-sectional studies using imaging, there is a lack of comprehensive understanding of the association between tumor glucose metabolism, differentiation and BRAF mutation. Meanwhile, recent comprehensive analyses using next generation sequencing have revealed new integrative molecular subtypes as well as cancer drivers [8]. In this regard, the integrative analysis of iodine and glucose metabolism based on the systemic gene expression data is needed for the molecular background of imaging and therapy of thyroid cancer.

The aim of this study is to identify the association between the glucose metabolism profile and the differentiation of thyroid cancer using transcriptomic signatures. As tumor glucose metabolism clinically assessed by FDG PET is associated with the expression of glucose transporters (GLUT) and glycolysis pathways [9–11], gene signatures of GLUT and glycolysis were used for defining glucose metabolic profiles of the tumors. We investigated whether the glucose metabolic profiles were correlated with tumor differentiation and BRAF mutations in advanced thyroid cancer as well as PTC. We also investigated whether these metabolic profiles were associated with clinical outcome, and eventually aimed to redefine the biological progression of thyroid cancer in terms of glucose metabolism as well as differentiation related with iodine uptake.

## Methods

### Subjects and data acquisition

RNA sequencing data of well differentiated papillary thyroid cancers (THCA) were obtained from The Cancer Genome Atlas (TCGA) which was publicly available. The gene expression data of 21,022 genes and clinical information from 505 patients with PTC were downloaded from the TCGA portal (http://portal.gd.cancer.gov/) using ‘TCGABiolinks’ R/Bioconductor package. Microarray data of poorly differentiated thyroid cancer (PDTC) and ATC were also obtained in NCBI’s Gene Expression Omnibus (GEO) Series accession number GSE76039. Clinical information of PDTC/ATC patients was downloaded from cBioPortal (http://www.cbioportal. GSE76039org/public-portal). Data from patients with PTC and PDTC/ATC were all collected from primary tumor, except 7 samples from recurred or metastatic tumors of PDTC/ATC (6 samples from recurred tumor, one sample from metastatic tumor).

### Glucose metabolic profiles

We normalized mRNA transcripts counts to adjust GC-content effects and between-lane distributional difference. Using normalized gene counts, we calculated the enrichment scores of glucose metabolic signatures for GLUT and glycolysis. Glycolysis signatures were defined by Reactome pathway [12]. To estimate the enrichment score of glycolysis, a single sample gene set enrichment analysis (ssGSEA) was performed using the curated gene sets of glycolysis signatures [13]. This analysis was performed with GSVA R/Bioconductor package [14]. For estimating GLUT score, the sum of log-expression values of GLUT1 and GLUT3 was used as FDG uptake is dependent on these two GLUT subtypes [15]. Tumor differentiation score (TDS) were also calculated by expression values of a set of specific genes [8]. Notably, the set of genes to calculate TDS did not include genes related to glucose transporters and glycolysis.

### Statistical analysis

Student’s t-test was used to analyze whether the signatures of glucose metabolism were significantly different according to the genetic mutation status of BRAFV600E. Pearson’s correlation test was performed to evaluate the associations between TDS and signatures of glucose metabolism. The difference between two correlation coefficients regarding BRAFV600E was calculated by Fisher z-transformation. In addition, the difference between two correlation coefficients according to the histologic cell types was calculated by Fisher z-transformation. The effect of the signatures of glucose metabolism on patients’ recurrence-free survival was analyzed using the Cox proportional hazard model. Patients were divided into two groups; lower or higher than the median value of GLUT, and glycolysis unit. Kaplan-Meier survival curves were demonstrated to compare recurrence-free survival between two groups. The statistical significance was evaluated using the Log rank test. The differences between groups were considered statistically significant at *p*-value < 0.05. All statistical analyses were performed with R 3.4.1 (https://www.r-project.org/).

## Results

To evaluate the association between tumor differentiation and glucose metabolism in the thyroid cancer, we used two cohorts, PTC samples of TCGA and PDTC/ATC samples obtained by GEO (GSE76039). The clinical and pathological characteristics of PTC patients and PDTC/ATC patients are summarized in Table 1 and Table 2. Notably, there were unavailable clinical data such as race.

**Table 1:** Clinicopathological characteristics of the 505 PTC included in the study

Variables	PTC	No. of available data
Gender (M:F)	136: 369	505
Age (years, mean ± SD)	46.89 ± 15.84 (15.38–89.54)	505
Race		
White / Asian / Black / Etc.	334 / 51 / 27 / 1	413
Histology		
Classical / Follicular / Tall cell / Other	323 / 99 / 34 / 9	465
BRAFV600E (Positive:Negative)	235: 270	505
Stage		
1 / 2 / 3 / 4	284 / 52 / 112 / 55	503
Recurrence-free survival time (months)	14.26 (0–158.71)	423

PTC Papillary Thyroid Cancer

**Data in parentheses are ranges**

**Table 2:** Clinicopathological characteristics of the 10 PDTC and 27 ATC included in the study

Variables	PDTC/ATC	No. of available data
Gender (M:F)	10: 27	37
Age (years, mean ± SD)	69 ± 13.74 (27–87)	37
Histology		
Papillary / Follicular / Tall cell / Other	14 / 5 / 5 / 5	29
BRAFV600E (Positive:Negative)	NA	
Stage		
1 / 2 or 3 / 4	1 / 5 / 24	30
Recurrence-free survival time (months)	10.3 (0.23–223.49)	35

PDTC/ATC Poorly-Differentiated Thyroid Cancer/Anaplastic Thyroid Cancer

**Data in parentheses are ranges**

**Fig. 1:**
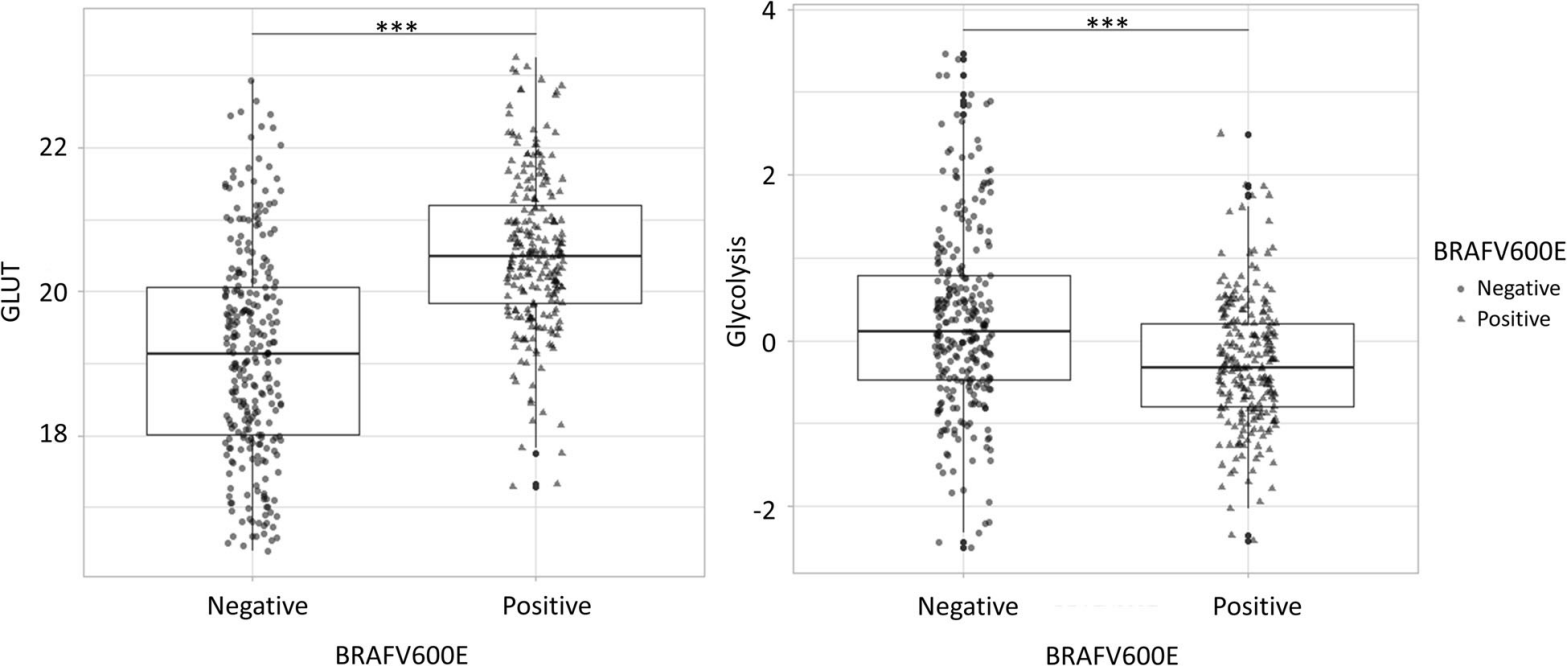
Box and whiskers plot of GLUT and glycolysis signatures by BRAFV600E mutation in PTC. The line across each box represent the median, and the top edge, and the bottom edge represents the first quartile, and the third quartile, respectively. Student’s t-test showed significant difference of signatures of GLUT and glycolysis, between BRAFV600E positive and negative groups (BRAFV600E positive group 20.53 ± 1.08 vs. BRAFV600E negative group 19.13 ± 1.46, t = − 12.09, *p* < 0.0001 for GLUT; BRAFV600E positive group − 0.28 ± 0.77 vs. BRAFV600E negative group 0.24 ± 1.11, t = 6.06, *p* < 0.0001 for glycolysis). (*** = *p* < 0.001)

Firstly, GLUT and glycolysis enrichment scores were estimated by gene expression data of the two cohorts and associated with the mutation status, BRAFV600E. The enrichment scores of GLUT and glycolysis were significantly different in PTC according to BRAFV600E mutation status (Fig. 1). BRAFV600E positive PTC have higher GLUT signature and lower glycolysis signature (BRAFV600E positive group 20.53 ± 1.08 vs. BRAFV600E negative group 19.13 ± 1.46, t = − 12.09, *p* < 0.0001 for GLUT; BRAFV600E positive group − 0.28 ± 0.77 vs. BRAFV600E negative group 0.24 ± 1.11, t = 6.06, *p* < 0.0001 for glycolysis) than BRAFV600E negative PTC (Fig. 1).

**Fig. 2:**
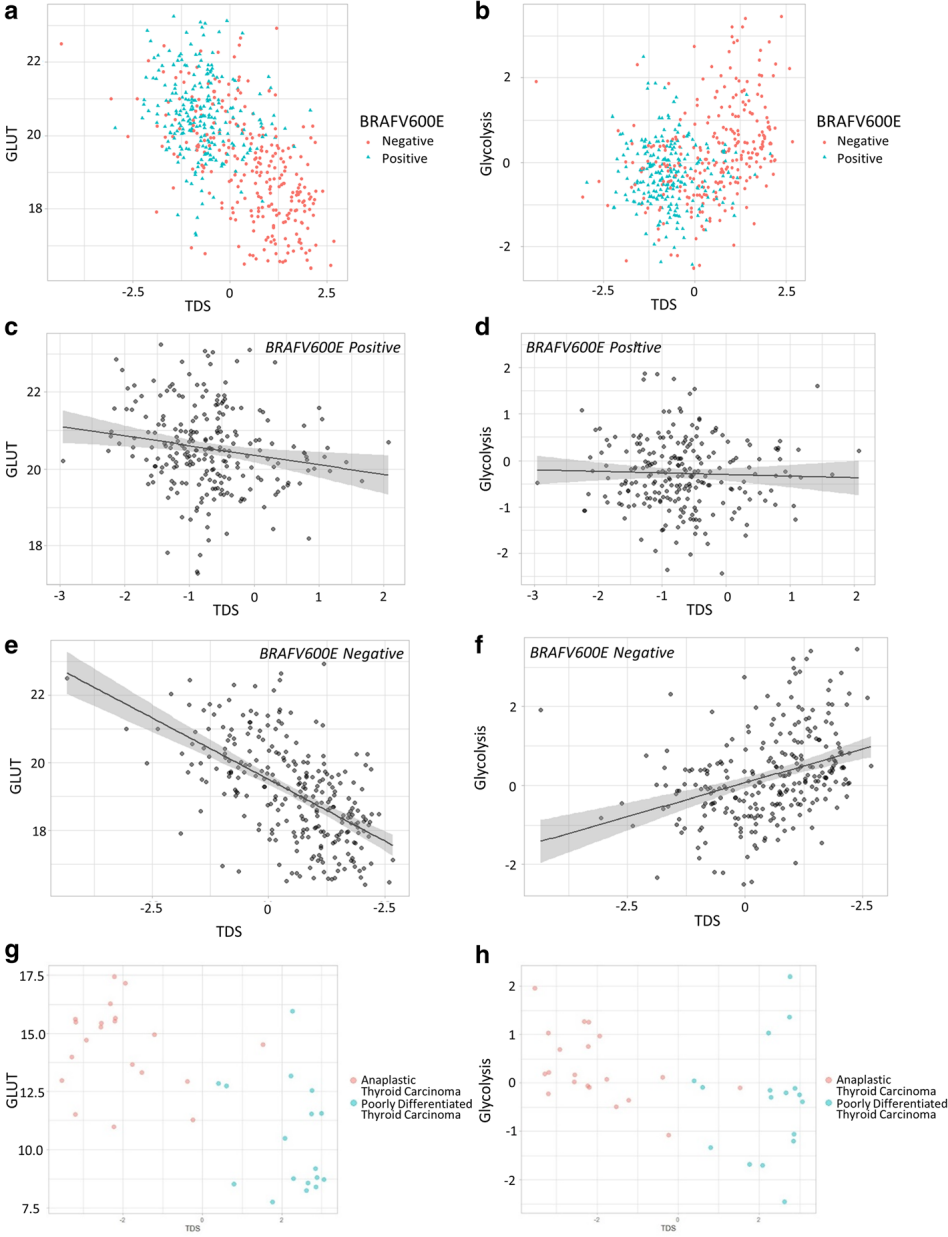
Scatter plot of TDS versus GLUT and glycolysis signatures in PTC and PDTC/ATC. (**a, b**) Pearson’s correlation analysis showed that negative correlation was shown between TDS and GLUT (r = − 0.59, *p* < 0.0001), while positive correlation was shown between TDS and glycolysis (r = 0.33, *p* < 0.0001). (**c, d**) GLUT and glycolysis signatures of BRAFV600E positive group showed no significant correlation with TDS (r = − 0.18, *p* = 0.065 and r = − 0.03, *p* = 0.605, respectively). (**e, f**) GLUT and glycolysis signatures of BRAFV600E negative group were significantly correlated with TDS in PTC (r = − 0.57, *p* < 0.001 and r = 0.35, *p* < 0.001, respectively). (**g, h**) Pearson’s correlation analysis showed that signatures of GLUT and glycolysis both have negative correlation with TDS in PDTC/ATC (r = − 0.68, *p* < 0.0001 and r = − 0.38, *p* = 0.019, respectively)

**Fig. 3:**
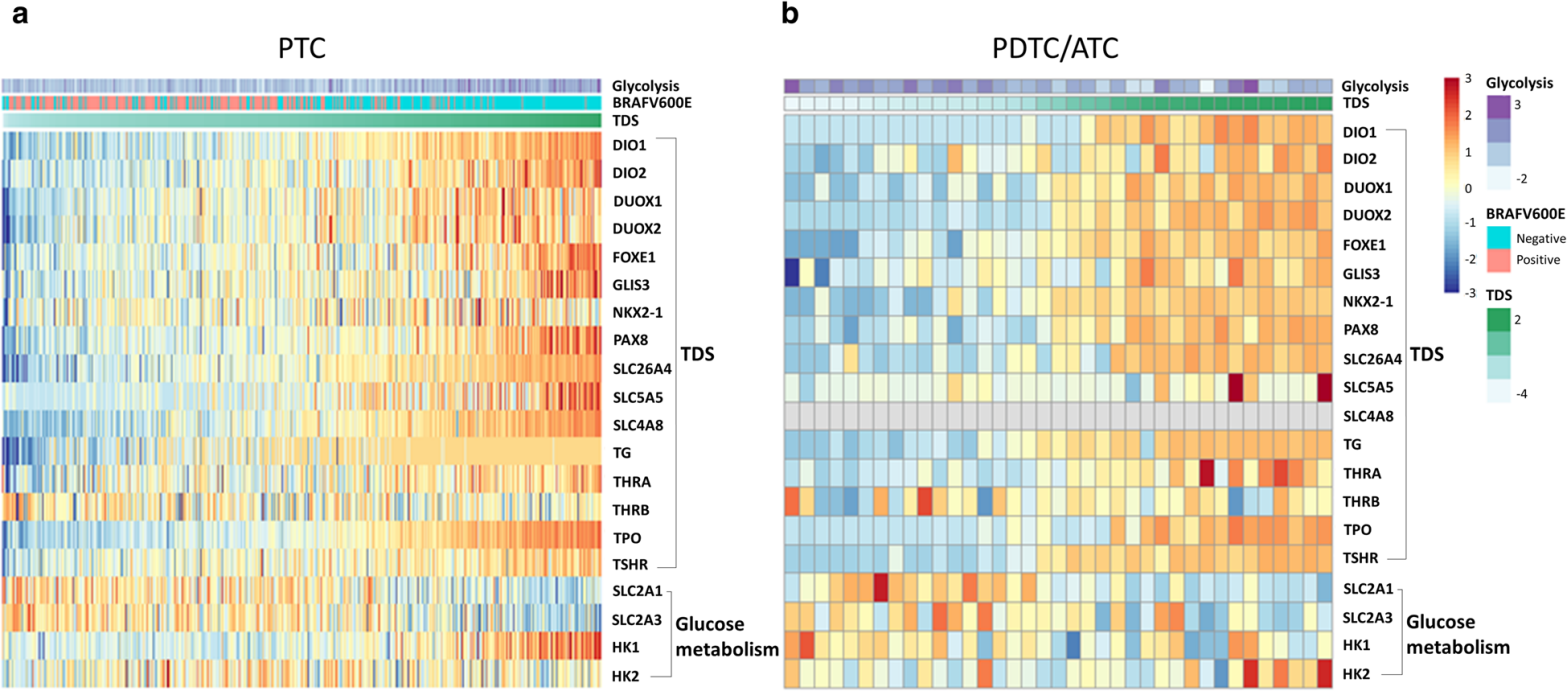
Heatmaps of genes related to glucose metabolism and TDS in PTC and PDTC/ATC. Glycolysis signature with TDS, BRAFV600E mutation status, and gene expression data for sixteen genes constituting TDS, two genes for GLUT (SLC2A1, SLC2A3), and two genes for hexokinase (HK) are shown in PTC (**a**) and PDTC/ATC (**b**)

To evaluate the tumor differentiation, we employed tumor differentiation score, TDS, calculated by sixteen genes related to thyroid functions. The TDS was compared with glucose metabolic profiles. In PTC group, the TDS was negatively correlated with GLUT signature, but positively correlated with glycolysis signature (r = − 0.59, *p* < 0.0001 for GLUT; Fig. 2a, r = 0.33, *p* < 0.0001 for glycolysis; Fig. 2b). We divided PTC into two subgroups according to the BRAFV600E mutation status. PTC without BRAFV600E showed a negative correlation between TDS and GLUT (r = − 0.57, *p* < 0.001), while it showed a positive correlation between TDS and glycolysis (r = 0.35, *p* < 0.001). PTC with BRAFV600E showed a trend of negative correlation between GLUT and TDS (r = − 0.18, *p* = 0.065) and no significant correlation between glycolysis and TDS. These correlations were significantly stronger in BRAFV600E negative group than positive group (z = 5.22 for GLUT, z = − 4.48 for glycolysis, and *p* < 0.001 for both GLUT and glycolysis; Fig. 2c-f). In PDTC/ATC group, the relationship between the TDS and GLUT signature showed a significant negative correlation (r = − 0.68, *p* < 0.0001; Fig. 2g). The TDS also showed a significant negative correlation to the glycolysis signature (r = − 0.38, *p* = 0.019; Fig. 2h). We also evaluated whether different cell types of PTC were associated with glucose metabolic signatures. Classical cell type PTC have higher GLUT signature and lower glycolysis signature than follicular cell type PTC (Classical cell type 20.13 ± 1.32 vs. Follicular cell type 18.42 ± 1.20, t = 11.55, *p* < 0.001 for GLUT; Classical cell type − 0.13 ± 0.94 vs. Follicular cell type 0.52 ± 1.12, t = − 5.65, *p* < 0.001 for glycolysis; Additional file 1: Figure S1A, B). The negative correlation between TDS and GLUT and the positive correlation between TDS and glycolysis were found in both cell types, classical and follicular types. The strength of their correlations showed no significant difference when we compare the correlation coefficients by Fisher z-transformation (Classical cell type: r = − 0.47, *p* < 0.001 for GLUT, r = 0.23, *p* < 0.001 for glycolysis; Follicular cell type: r = − 0.43, *p* < 0.001 for GLUT, r = 0.34, *p* = 0.001 for glycolysis; Additional file 1: Figure S1C, D). Individual genes that constitute TDS and glucose metabolic pathway were represented by heatmaps (Fig. 3).

**Fig. 4:**
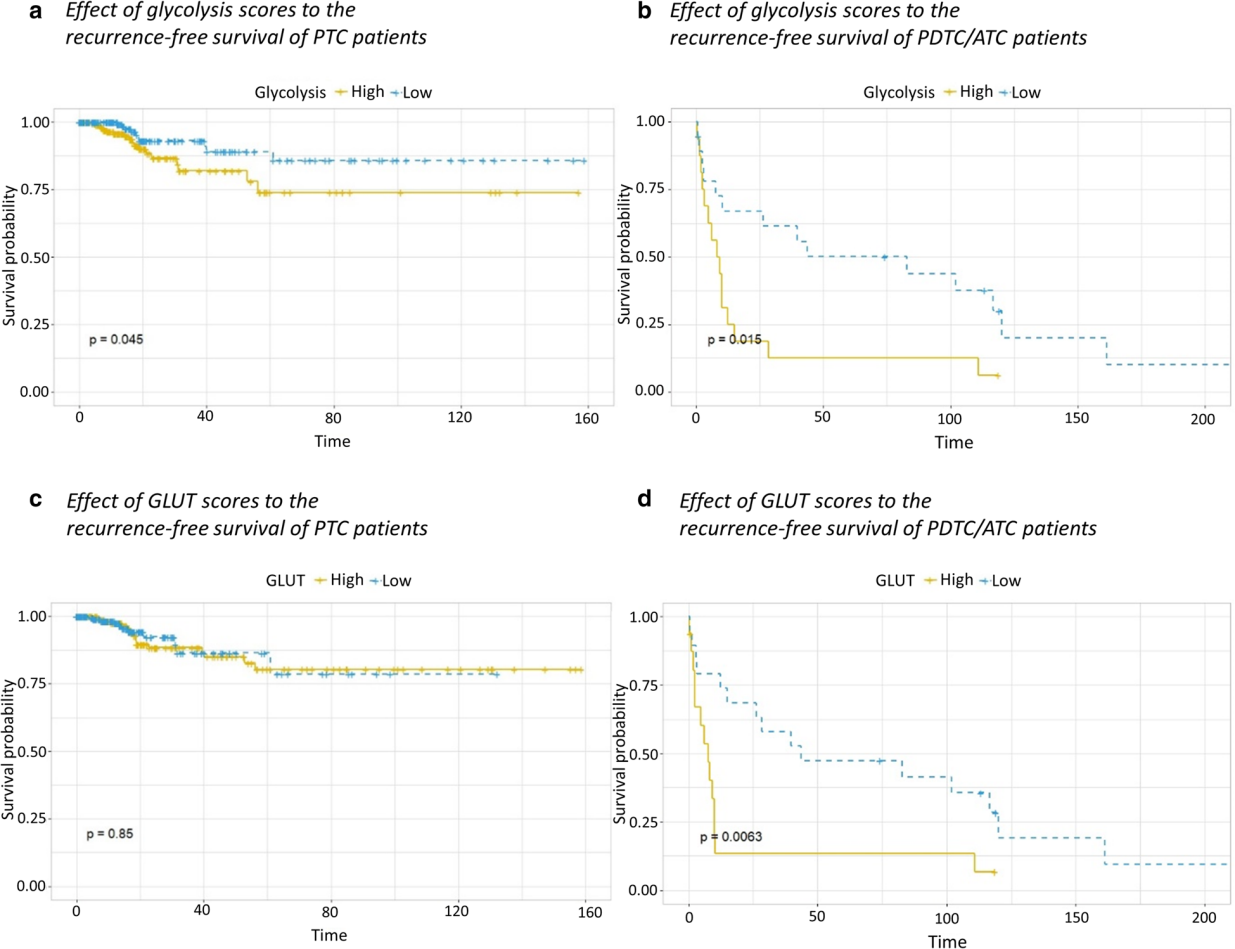
The Kaplan-Meier survival curve for recurrence-free survival of thyroid cancer patients. The patients were divided into two groups, based on the median value of each signature of glucose metabolism. PTC patients with low glycolysis signature have significantly less recurrence than the other group (**a**, *p* = 0.045). PDTC/ATC patients with low glycolysis signature showed better prognosis (**b**, *p* = 0.015). GLUT signature showed no significant correlation with recurrence-free survival in PTC patients (**c**, *p* = 0.85). PDTC/ATC patients with low GLUT signature showed better prognosis (**d**, *p* = 0.0063)

We assessed the association between signatures of glucose metabolism and patients’ prognosis. Kaplan-Meier survival curves of both PTC and PDTC/ATC patients with low glycolysis signature showed significantly better recurrence-free survival than the other group (*p* = 0.045 and 0.015, respectively, estimated by log-rank tests; Fig. 4a, b). The glycolysis signature of the primary tumor showed a significant correlation with the N-stage (N negative group 0.13 ± 1.03 vs. N positive group − 0.24 ± 0.89, t = 3.86, *p* = 0.0001), but not with the M-stage (M negative group − 0.08 ± 1.03 vs. M positive group − 0.16 ± 0.89, t = 0.22, *p* = 0.823; Additional file 2: Figure S2). We then assessed the predictive value of glycolysis signature for recurrence using a cox proportional hazard model. The hazard ratios (H.R.) of the influence of the glycolysis signature on the recurrence of PTC are estimated by uni- and multi-variate analyses (Table 3). In PTC, a univariate cox hazard proportional model revealed that the high glycolysis signature alone had a significant influence on the recurrence-free survival (H.R. = 1.50; C.I. = 1.03–2.17; *p* = 0.033). The adjustment for age and gender maintained its influence on the recurrence-free survival (H.R. = 1.50; C.I. = 1.04–2.16; *p* = 0.031). Additional adjustment for T-stage and N-stage still demonstrated worse prognosis on PTC patients with high glycolysis (H.R. = 1.92; C.I. = 1.22–3.00; *p* = 0.005). Although TDS itself did not influence the recurrence-free survival (H.R. = 0.76; C.I. = 0.55–1.06; *p* = 0.102), the adjustment for TDS as well as age, gender, T-stage, and N-stage maintained the influence of glycolysis signature on the recurrence-free survival (H.R. = 1.98; C.I. = 1.26–3.10; *p* = 0.003). The clinicopathological characteristics between PTC patients with low glycolysis and PTC patients with high glycolysis showed no significant difference except for age (Table 4). GLUT score showed no significant correlation with PTC patients’ prognosis (*p* = 0.85; Fig. 4c). On the contrary, we found that PDTC/ATC patients with low GLUT signature have significantly longer recurrence-free survival than the other group (*p* = 0.0063; Fig. 4d).

**Table 3:** Univariate and multivariate analysis of influence on the recurrence of PTC

Variables *(The first group is the reference group)*	Univariate analysis		Multivariate analysis	
	H.R. (95% C.I.)	*p*-value	H.R. (95% C.I.)	*p*-value
Age (Continuous variable)	1.01 (0.99–1.04)	0.351	1.01 (0.99–1.04)	0.265
Gender (Female vs. Male)	1.56 (0.71–3.45)	0.272	1.98 (0.83–4.71)	0.124
T-stage (T1 + T2 vs. ≥T3)	1.36 (0.63–2.95)	0.435	1.41 (0.61–3.21)	0.420
N-stage (N0 vs. ≥N1)	3.76 (1.40–10.09)	0.008	4.24 (1.50–11.96)	0.006
TDS (Continuous variable)	0.76 (0.55–1.06)	0.102	0.83 (0.58–1.18)	0.293
Glycolysis signature (Continuous variable)	1.50 (1.03–2.17)	0.033	1.98 (1.26–3.10)	0.003

PTC Papillary Thyroid Cancer; H.R Hazard Ratio; C.I Confidence Interval; TDS Tumor Differentiation Score

**Table 4:** Clinicopathological characteristics of the 423 PTC

Variables	PTCs with low glycolysis signature	PTCs with high glycolysis signature	χ^2*^ or t^†^ or H.R.^§^/ *p*-value
Gender (M:F)	59:153	50:161	0.74^*^/0.389
Age (years)	43 (15–89)	47 (19–83)	3.06^†^/0.002
Stage			
1 / 2 / 3 / 4	131 / 15 / 45 / 20	119 / 23 / 48 / 20	0.995^‡^
Recurrence-free survival time (months)	14.59 (0–158.71)	14.06 (0–157.03)	1.50^§^/0.033

^‡^ *p*-value from Analysis of Variance

PTC Papillary Thyroid Cancer; H.R Hazard Ratio

**Data in parentheses are ranges**

## Discussion

In this study, we used transcriptome data of thyroid cancers of two cohorts, PTC and PDTC/ATC to evaluate glucose metabolic profiles and tumor differentiation. In PTC, a trend of higher GLUT and lower glycolysis was found in tumors with BRAFV600E mutation and those with relatively poor differentiation. There is a controversy regarding the correlation of BRAFV600E, GLUT1, and tumor differentiation genes according to several previous reports [16–18]. Our results are consistent with other results of higher GLUT1 in less differentiated thyroid cancer [16] and negative correlation between BRAFV600E mutation and tumor differentiation genes in PTC [17, 18]. The results of TDS positively correlated with glycolysis and negatively correlated with GLUT were particularly found in PTC without BRAFV600E mutation. Moreover, the high glycolysis enrichment was significantly associated with poor clinical outcome, even it was associated with well differentiation in PTC. However, this paradoxical opposite direction of correlation was not found in PDTC/ATC cohorts, which showed both GLUT and glycolysis were negatively correlated with TDS and associated with poor clinical outcome.

We have found that thyroid cancers with poor differentiation showed higher GLUT expression (Fig. 2). Cancer cells demand a higher amount of energy according to the progression, which is associated with enhanced aerobic glycolysis in the advanced cancer [19]. The glucose demand of cancers cause overexpression of GLUT1 and/or GLUT3 to increase glucose influx [20]. Our results were compatible with the previous studies since poorly differentiated thyroid cancers would need higher glucose uptake through GLUT expression [21]. On the other hand, glycolysis signatures of thyroid cancers with poor differentiation were inconsistent between PTC and PDTC/ATC (Fig. 2). In PTC, relatively well-differentiated tumors, glycolysis was positively associated with TDS. In PDTC/ATC, a negative correlation was shown between TDS and glycolysis. Considering PTC of TCGA data are relatively well-differentiated tumors compared with PDTC/ATC cohort, the association between the differentiation and glycolysis might have ‘U shape’ pattern; high glycolysis signatures were shown in ATC and some types of well-differentiated PTC. Moreover, increased glycolysis was associated with poor clinical outcome in spite of high TDS. It implies that the differentiation of thyroid cancer may not be the only factor that reflects the biological progression of thyroid cancer. Instead, in addition to differentiation, glucose metabolic profiles represented by glycolysis should be further considered to infer the progression of thyroid cancer, particularly for the specific subtypes of PTC, BRAFV600E negative and/or follicular variants.

Our results demonstrated that the signatures of GLUT and glycolysis can act as prognostic factors in predicting recurrence-free survival of thyroid cancer patients (Fig. 4). The multivariate analysis revealed that the two variables, N-stage and the glycolysis signature, were significantly associated with the recurrence. As N-stage has been regarded as an important conventional biomarker related to prognosis which can be confirmed by surgical exploration, our results emphasized again the importance of the lymph node status in thyroid cancer as postoperative risk stratification. In terms of another prognostic marker in our results, glycolysis signature, it is notable that glucose metabolism profiles can be noninvasively estimated by FDG PET. GLUT score, calculated by GLUT1 and GLUT3, was reported as the major deterministic factor for the FDG uptake in various studies [10, 15, 21, 22], while a recent study showed a moderate correlation between FDG uptake and GLUT regarding a complex mechanism of glucose metabolism [23]. Furthermore, glycolysis activity is also associated with FDG uptake in vivo [22, 24]. According to the kinetic model of FDG, glycolysis activity is associated with FDG retention, which can be visualized by dual-time FDG PET [25, 26]. In general, poorly differentiated thyroid cancers are known to have a worse outcome as compared to well differentiated thyroid cancers [27]. However, a subset of well-differentiated carcinoma shows relatively poorly outcome in tumors with increased glycolysis with well-differentiated type. As GLUT and glycolytic activity were differently associated with TDS, noninvasive characterization using FDG PET and radioactive iodine imaging could play a role in risk stratification when considered with other prognostic factors as well as biological characterization of the tumor. As a future work, more specifically, dual-time FDG PET could be used for estimating glycolysis activity, which might identify a subtype of the tumor with enhanced glycolysis and well-differentiation.

Although, we analyzed glucose profiles of thyroid cancer in the two different cohorts with different differentiation, there are some limitations. Firstly, the PDTC/ATC sample size was small. Though we found the expected role of GLUT and glycolysis on the prognosis of PDTC/ATC patients, further studies with a larger group are needed. Another limitation was that the transcriptome data from two cohorts were not be combined and analyzed since those data were obtained from different resources, RNA sequencing and microarray. For PDTC/ATC patients, a few samples were acquired from recurred or metastatic tumors (recurred tumors for 6 samples and one sample from metastatic tumor), which might affect our analysis though recurred and metastatic tumors have similar glucose metabolic profiles with their primary tumors. Furthermore, noninvasive imaging biomarkers using iodine scan and FDG PET integrated with transcriptomic data could clarify our results of the association of differentiation and glucose metabolic profiles. We could expect the clinical application of our results as a future study, such as the estimation of glycolysis score based on gene expression profiles in fine-needle aspiration samples. In addition, according to our results, the expression of genes related to glycolysis may be examined by tissue samples in the clinic to stratify patients’ outcome, even though further studies focusing on clinical outcomes and clinical decision according to the glycolysis are needed.

## Conclusions

According to the integrative analysis of iodine and glucose metabolism based on the systemic gene expression data, metabolic profiles were not simply associated with tumor differentiation. Cancer cellular GLUT expression was negatively associated with tumor differentiation in both PTC and PDTC/ATC. The enrichment of glycolysis was positively associated with the differentiation in well-differentiated PTC, while it was negatively associated with the PDTC/ATC. Overall, there might be ‘U-shape’ pattern for the association of the differentiation and glycolysis. Furthermore, increased glycolysis was poor prognosis in spite of the well-differentiated tumor. We anticipate that the biological and prognostic characteristics of glucose metabolic profiles could provide insight for biomarker using FDG PET and appropriate therapeutic plan.

## Supplementary information

**Additional file 1 Figure S1.** Plots for GLUT and glycolysis signatures in PTC with different cell types. (A) Box and whiskers plot of GLUT signature in PTC according to cell type (Classical cell type 20.13 ± 1.32 vs. Follicular cell type 18.42 ± 1.20, t = 11.55, *p* < 0.001) (B) Box and whiskers plot of glycolysis signature in PTC according to cell type (Classical cell type − 0.13 ± 0.94 vs. Follicular cell type 0.52 ± 1.12, t = − 5.65, *p* < 0.001) (C) Scatter plot of TDS versus glucose metabolism signature in classical cell type PTC (r = − 0.47, *p* < 0.001 for GLUT; r = 0.23, *p* < 0.001 for glycolysis) (D) Scatter plot of TDS versus glucose metabolism signature in follicular cell type PTC (r = 0.43, *p* < 0.001 for GLUT; r = 0.34, *p* = 0.001 for glycolysis)

**Additional file 2 Figure S2.** Box and whisker plot of glycolysis signatures by N-stage or M-stage in PTC. The line across each box represent the median, and the top edge, and the bottom edge represents the first quartile, and the third quartile, respectively. Student’s t-test showed significant difference of signatures of glycolysis between N positive and N negative groups (N negative group 0.13 ± 1.03 vs. N positive group − 0.24 ± 0.89, t = 3.86, *p* = 0.0001). No significant difference of signatures of glycolysis were found between M positive and M negative groups (M negative group − 0.08 ± 1.03 vs. M positive group − 0.16 ± 0.89, t = 0.22, *p* = 0.82). (*** = *p* < 0.001)

## Data Availability

The datasets used and/or analyzed during the current study are available in the TCGA Research Networks: http://cancergenome.nih.gov/ and GEO: http://www.ncbi.nlm.nih.gov/geo.

## Abbreviations

ATC: Anaplastic thyroid cancer
BRAF: B-type Raf kinase
C.I.: Confidence Interval
FDG: 2-deoxy-2-[18F]fluoro-D-glucose
GEO: Gene Expression Omnibus
GLUT: Glucose transporter
H.R: Hazard ratio
HK: Hexokinase
PDTC: Poorly differentiated thyroid cancer
PET: Positron emission tomography
PTC: Papillary thyroid cancer
ssGSEA: Single sample gene set enrichment analysis
TCGA: The Cancer Genome Atlas
THCA: Well differentiated papillary thyroid cancer
TSD: Tumor differentiation score
