# Supplementary figures and images for: Comprehensive gene expression analysis for exploring the association between glucose metabolism and differentiation of thyroid cancer
Source: BMC Cancer. 2019 Dec 30;19:1260. doi: 10.1186/s12885-019-6482-7 (PMC6937781; doi:10.1186/s12885-019-6482-7)

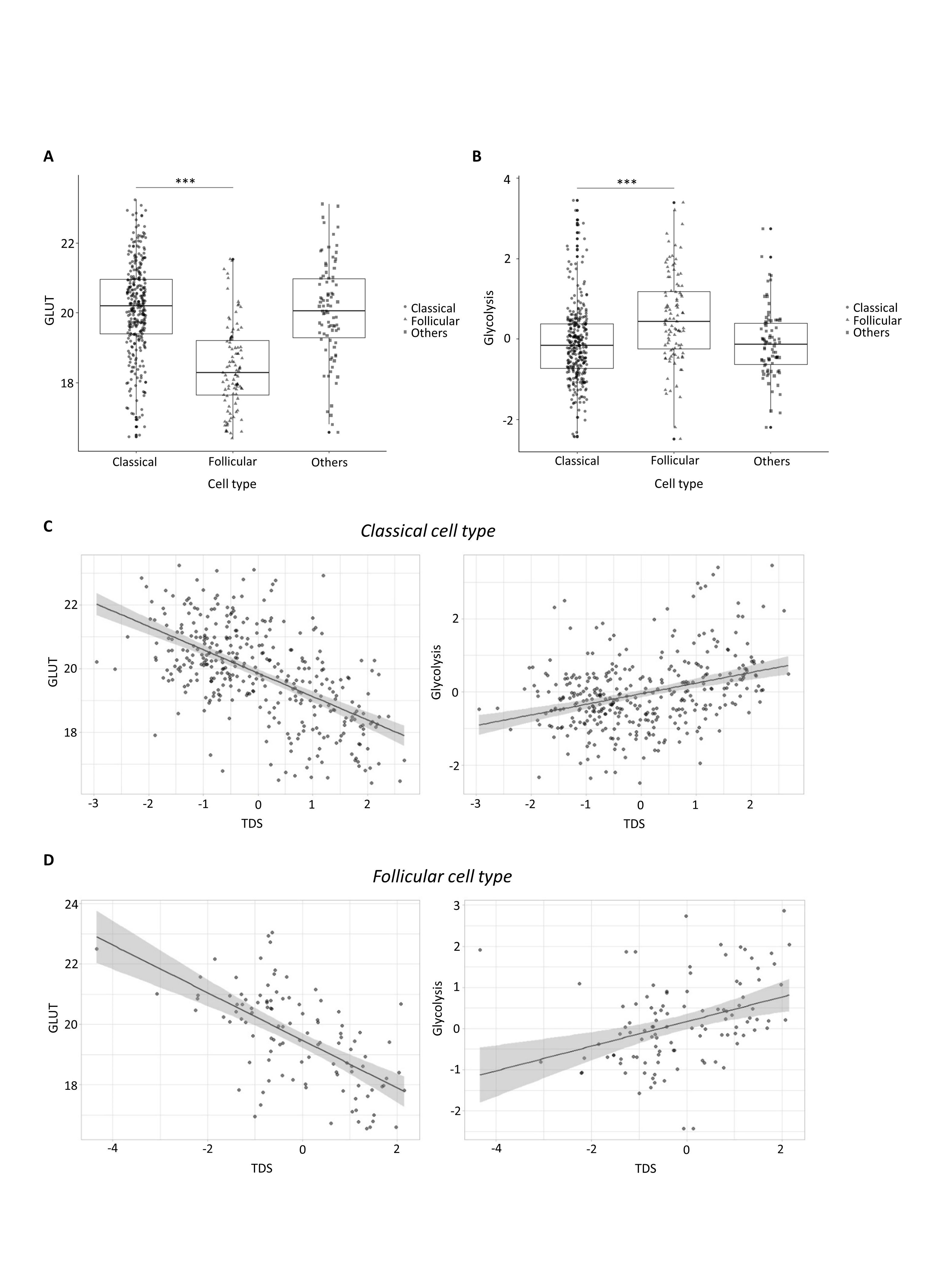

Supplement: Supplementary file 1 — Additional file 1 Figure S1. Plots for GLUT and glycolysis signatures in PTC with different cell types. (A) Box and whiskers plot of GLUT signature in PTC according to cell type (Classical cell type 20.13 ± 1.32 vs. Follicular cell type 18.42 ± 1.20, t = 11.55, p < 0.001) (B) Box and whiskers plot of glycolysis signature in PTC according to cell type (Classical cell type − 0.13 ± 0.94 vs. Follicular cell type 0.52 ± 1.12, t = − 5.65, p < 0.001) (C) Scatter plot of TDS versus glucose metabolism signature in classical cell type PTC (r = − 0.47, p < 0.001 for GLUT; r = 0.23, p < 0.001 for glycolysis) (D) Scatter plot of TDS versus glucose metabolism signature in follicular cell type PTC (r = 0.43, p < 0.001 for GLUT; r = 0.34, p = 0.001 for glycolysis) [file 12885_2019_6482_MOESM1_ESM.tif]

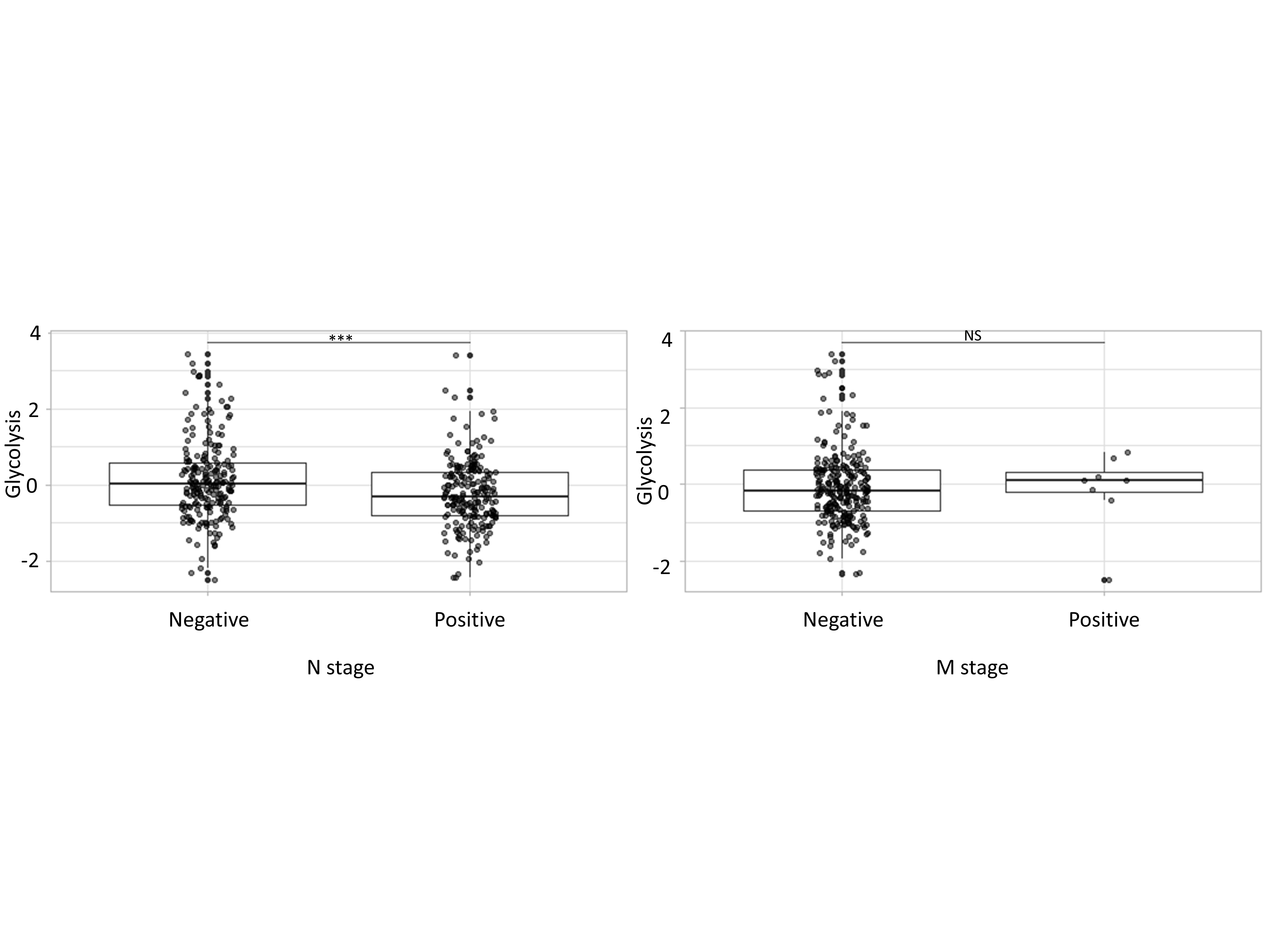

Supplement: Supplementary file 2 — Additional file 2 Figure S2. Box and whisker plot of glycolysis signatures by N-stage or M-stage in PTC. The line across each box represent the median, and the top edge, and the bottom edge represents the first quartile, and the third quartile, respectively. Student’s t-test showed significant difference of signatures of glycolysis between N positive and N negative groups (N negative group 0.13 ± 1.03 vs. N positive group − 0.24 ± 0.89, t = 3.86, p = 0.0001). No significant difference of signatures of glycolysis were found between M positive and M negative groups (M negative group − 0.08 ± 1.03 vs. M positive group − 0.16 ± 0.89, t = 0.22, p = 0.82). (*** = p < 0.001) [file 12885_2019_6482_MOESM2_ESM.tif]
